# Obesity Is Not Associated With an Increased Risk of Serious Infections in Biologic-Treated Patients With Inflammatory Bowel Diseases

**DOI:** 10.14309/ctg.0000000000000380

**Published:** 2021-07-06

**Authors:** Siddharth Singh, Herbert C. Heien, Lindsey Sangaralingham, Nilay D. Shah, William J. Sandborn

**Affiliations:** 1 Division of Gastroenterology, University of California San Diego, La Jolla, California, USA;; 2 Division of Biomedical Informatics, Department of Medicine, University of California San Diego, La Jolla, California, USA;; 3 Robert D. and Patricia E. Kern Center for the Science of Health Care Delivery, Mayo Clinic, Rochester, Minnesota, USA;; 4 Division of Health Care Policy and Research, Department of Health Services Research, Mayo Clinic, Rochester, Minnesota, USA.

## Abstract

**INTRODUCTION:**

Obesity has been associated with adverse disease-related outcomes and inferior treatment response to biologic agents in patients with inflammatory bowel diseases (IBDs), but its impact on the risk of treatment-related complications is unknown. We performed a cohort study examining the association between obesity and risk of serious infections in biologic-treated patients with IBD.

**METHODS:**

Using an administrative claims database, in a cohort of biologic-treated patients with IBD between 2014 and 2018 with follow-up 1 year before and after treatment initiation, we compared the risk of serious infections (infections requiring hospitalization) between obese vs nonobese patients (based on validated administrative claims) using Cox proportional hazard analysis.

**RESULTS:**

We included 5,987 biologic-treated patients with IBD (4,881 on tumor necrosis factor-α antagonists and 1,106 on vedolizumab), of whom 524 (8.8%) were classified as obese. Of the 7,115 person-year follow-up, 520 patients developed serious infection. Risk of serious infection was comparable in obese vs nonobese patients (8.8% vs 8.5%; unadjusted hazard ratio, 1.15; 95% confidence interval, 0.86–1.54). After adjusting for age, comorbidities, disease characteristics, health care utilization, use of corticosteroids, immunomodulators, and opiates, obesity was not associated with an increased risk of serious infection (adjusted hazard ratio, 0.74 [95% confidence interval, 0.55–1.01]). Similar results were seen on stratified analysis by disease phenotype (Crohn's disease and ulcerative colitis) and index biologic therapy (tumor necrosis factor-α antagonists and vedolizumab).

**DISCUSSION:**

After adjusting for comorbid conditions and disease characteristics, obesity is not independently associated with an increased risk of serious infections in biologic-treated patients with IBD.

## INTRODUCTION

Approximately 10%–35% of patients with inflammatory bowel diseases (IBDs) are obese, and an additional 20%–40% may be overweight (1). Obesity has been associated with adverse outcomes in patients with IBD, including higher risk of clinical relapse, inferior quality of life, higher burden and costs of hospitalization, and potentially higher risk of surgery (1–4). Obesity has also been variably associated with inferior response to therapy, particularly with biologic agents, and a lower likelihood of achieving endoscopic remission (4–6). Although this negative impact of obesity on disease-related complications in patients with IBD has been established, there has been limited evaluation of the impact of obesity on the risk of treatment-related complications, particularly risk of serious infections.

Fat, particularly visceral adipose tissue, is metabolically active and can modify the immune response through numerous proinflammatory and anti-inflammatory adipokines, cytokines, and chemokines, which can predispose to infection (7). Obesity has been consistently associated with an increased risk of surgical site infections (8). More recently, the negative impact of obesity on risk and outcomes of coronavirus 2019 has been established (9). However, whether obesity modifies the risk of infections in patients with IBD, particularly those being treated with immunosuppressive agents, has not been well studied. Registry studies of biologic agents, including infliximab (TREAT, ENCORE) and adalimumab (PYRAMID), and open-label extension safety studies of vedolizumab and ustekinumab have not examined whether obesity is a risk factor for serious infections (10–16). In an open-label extension study of tofacitinib in ulcerative colitis, higher body weight was associated with an increased risk of serious infections (17). Real-world studies have identified that multimorbidity is independently associated with an increased risk of serious infection; however, they have not examined the impact of obesity on the risk of infections (18,19).

Hence, to understand the association between obesity and risk of serious infections in biologic-treated patients with IBD, we conducted a retrospective cohort study using a large deidentified administrative claims database.

## METHODS

### Data source

We conducted a retrospective analysis of deidentified medical and pharmacy administrative claims from a large database (Data Warehouse; OptumLabs, Cambridge, MA), which includes commercially insured and Medicare Advantage enrollees throughout the United States (20). The database contains data on more than 100 million enrollees from geographically diverse regions across the United States, with the greatest representation from the South and Midwest. Medical claims include *International Classification of Diseases, Ninth Revision and Tenth Revision, Clinical Modification* (*ICD-9-CM*; *ICD-10-CM*) diagnostic codes; *ICD-9* and *ICD-10* procedure codes; *Current Procedural Terminology, Fourth Edition* procedure codes; Healthcare Common Procedure Coding System procedure codes; site of service codes; and provider specialty codes. All study data were accessed using techniques compliant with the Health Insurance Portability and Accountability Act of 1996, and because this study involved analysis of pre-existing deidentified data, it was exempt from institutional review board approval.

### Study population

Between January 1, 2014, and December 31, 2018, we identified patients who filled a prescription or received an infusion for tumor necrosis factor α (TNFα) antagonists (infliximab, adalimumab, certolizumab pegol, and/or golimumab) and/or vedolizumab. From this cohort, we included adult patients (18–89 years) with (i) at least 1 diagnostic code for IBD (Crohn's disease [CD]: *ICD-9* 555.x or *ICD-10* K50; ulcerative colitis [UC]: *ICD-9* 556.x or *ICD-10* K51) before index date for receipt of candidate biologic agent, either from an inpatient or from an outpatient visit and (ii) continuous health plan enrollment with pharmacy benefits, with no prescription for candidate biologic in the 12 months before index date (new-user design), and minimum 12-month enrollment in health plan after index date. Patients who received candidate agent for <12 months, and discontinued due to intolerance or nonresponse, but still remained in the health plan were included. If a patient received diagnostic codes for both CD and UC, the patient was classified as having CD if most diagnostic codes were for CD. We excluded patients with (i) HIV infection, congenital immunodeficiency, or organ transplantation, or (ii) concomitant diagnosis of rheumatoid arthritis, ankylosing spondylitis, psoriasis, or psoriatic arthritis within the baseline 12-month period before prescription of TNFα antagonists.

### Exposure

Patients were classified as obese at the time of initiating biologic if they received *ICD-9* codes 278.00, 278.01, or V85.30-V85.44, or *ICD-10* code E66.x, any time during the 12 months preceding biologic initiation in an inpatient or outpatient encounter. These codes for obesity have been used in prior administrative database studies and demonstrated reliable accuracy, with high specificity and moderate sensitivity (21). In a large tertiary care center, detection of morbid obesity using *ICD-9* codes (V85.4, 278.01) when compared with chart-documented body mass index resulted in a sensitivity, a specificity, and positive and negative predictive values of 0.54, 0.99, and 0.82 and 0.96, respectively (21).

### Outcome

The primary outcome of interest was time to serious infections, defined as infection requiring hospitalization. These infections were identified based on principal discharge diagnoses (*ICD-9* or *ICD-10* codes) and included infections of the respiratory tract, skin and soft tissue, genitourinary tract, gastrointestinal tract, central nervous system, and septicemia/sepsis (22,23). Our definitions for serious infection requiring hospitalization have consistently shown positive predictive values of 80% or higher in previous studies, which considered medical chart reviews. We opted to focus only on hospitalized infection because these infections are severe and are significantly more likely to have adverse outcomes—including treatment discontinuation. By contrast, there is considerably more heterogeneity in severity of outpatient infections.

### Covariates

Baseline covariates (at the time of biologic exposure or in the preceding 12 months) included age, sex, race (gathered routinely by the database used), census region, calendar year, comorbidity burden measured using the Elixhauser index (12-month baseline period), unplanned health care utilization (defined as all-cause inpatient hospitalization or emergency department visits in the 12-month baseline period for each exposure), prior serious infections (12-month baseline period), as well as IBD phenotype (CD or UC), abdominal surgery (12-month baseline period), and receipt of endoscopy and/or abdominal imaging (12-month baseline period). We did not have access to individual patient medical records, endoscopy reports, or biochemical parameters.

### Statistical analysis

We used descriptive statistics to compare baseline demographic, clinical, and treatment characteristics in obese vs nonobese patients with IBD. We used the Pearson χ^2^ test to analyze categorical variables and the Student *t* test for continuous variables. Categorical variables are expressed as percentages and continuous variables as median with an interquartile range. All hypothesis testing was performed using a 2-sided *P* value with a statistical significance threshold of <0.05.

We performed survival analysis, using Kaplan-Meier curves, to evaluate the association between obesity and risk of serious infections in all biologic-treated patients with IBD, and by subgroups (disease phenotype: CD vs UC; age at the time of biologic initiation: ≤60 and >60 years; index biologic: TNFα antagonists vs vedolizumab; and comorbidity burden: Elixhauser index score <2 vs ≥2). Subsequently, to evaluate the independent effect of obesity on the risk of serious infections, we performed multivariable Cox proportional hazard analysis using backward variable selection, adjusting for age, sex, race/ethnicity, disease phenotype, index biologic agent, comorbidity burden, abdominal surgery, prior hospitalization or emergency department (ED) visit in baseline 12 months, prior infection in baseline 12 months, as well as use of corticosteroids, immunomodulators, and/or opiates within 3 months (and 12 months), before biologic initiation. All statistical analyses were performed with Stata MP (Stata Statistical Software: Release 14; StataCorp LP, College Station, Texas).

### Data availability statement

The data underlying this article were provided with permission by OptumLabs. Data will be shared on request to the corresponding author with permission of OptumLabs.

## RESULTS

Our cohort included 5,987 patients with IBD who were new users of TNFα antagonists or vedolizumab, of whom 524 (8.8%) were classified as obese based on diagnostic codes in the 12 months before the initiation of biologics. Baseline characteristics of patients classified as obese vs nonobese are shown in Table 1. Overall, obese patients were slightly older at biologic initiation (obese vs nonobese: 47 vs 41 years) and had higher rates of ED visits (60% vs 47%), hospitalization (39% vs 28%), prior serious infections (12% vs 7%), and higher burden of comorbidities (Elixhauser index score, 4 or more: 44% vs 13%).

**Table 1.** Baseline demographic characteristics, health care utilization, and IBD-related medication use in the 12 months before the initiation of index biologic, in obese vs nonobese patients with IBD

Variable	Obese (n = 524)	Nonobese (n = 5,463)
Demographic variables		
Mean age ± SD, yr	47 ± 14	41 ± 15
Sex (% males)	39.5	51.5
Race/ethnicity (%) White African American Others	69.9 14.7 15.4	72.5 11.9 15.6
IBD phenotype Crohn's disease (%) Ulcerative colitis (%)	55.3 44.7	58.3 41.7
Mean (±SD) follow-up after starting biologic (in mo)	13.6 ± 11.5	14.6 ± 13.5
Health care utilization and comorbidities (baseline 12 mo before biologic initiation)		
Emergency department visits (% of pts with ≥1)	59.7	46.7
Hospitalization (% of pts with ≥1)	38.8	28.0
Abdominal imaging (% of pts with ≥1)	60.3	54.0
Endoscopic procedures (% of pts with ≥1)	79.4	73.8
Abdominal surgery (% of pts with ≥1)	12.9	11.4
Mean (±SD) Elixhauser score Elixhauser score 2–3 Elixhauser score 4 or more	3.8 ± 2.5 40.6 44.1	1.6 ± 1.8 28.7 13.4
Major comorbidities Chronic obstructive lung disease Diabetes with or without complication Hypertension with or without complication Anemia	20.2 33.6 54.4 27.8	11.9 7.7 19.8 24.5
Serious infection (% of pts with ≥1)	12.1	7.5
IBD-related medication use		
TNFα antagonists (at index date) (%)	74.5	78.4
Vedolizumab (at index date) (%)	21.9	17.3
Recent immunomodulator use (within 3 mo before biologic initiation)	14.3	17.2
Oral corticosteroids (in baseline 12 mo), %	78.1	74.1
Opiates (in baseline 12 mo), %	53.7	40.9

IBD, inflammatory bowel disease; pts, patients; TNF, tumor necrosis factor.

**Figure 1.**
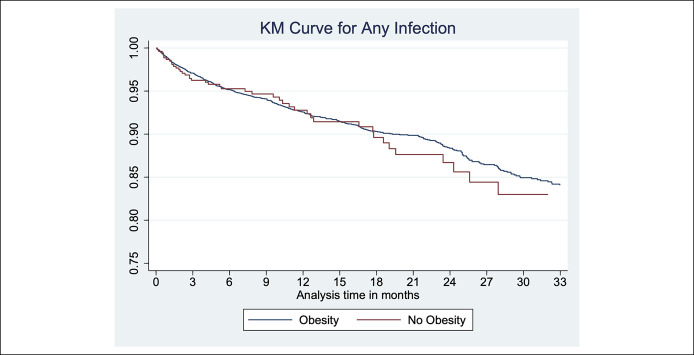
Risk of serious infection in obese vs nonobese patients in biologic-treated patients with inflammatory bowel disease. KM, Kaplan-Meier.

On follow-up, over 7,115 person-year follow-up, 520 patients developed serious infection. Systemic infections including sepsis, gastrointestinal, and pulmonary infections were most common. On univariate analysis, risk of serious infections was comparable in obese vs nonobese patients (8.8% vs 8.5%; unadjusted HR, 1.15; 95% confidence interval, 0.86–1.54) (Table 2, Figure 1). This risk of serious infections in obese vs nonobese patients was similar in several strata, including disease phenotype (CD and UC), age (≤60 and >60 years), type of biologic exposure (TNFα antagonists and vedolizumab), and in patients with or without major comorbidities (Table 2).

**Table 2.** Association between obesity and risk of serious infections in biologic-treated patients with inflammatory bowel diseases, overall and by predefined strata, on univariate analysis and multivariate analysis (adjusted for age, comorbidities, disease characteristics, health care utilization, use of corticosteroids, immunomodulators, and opiates)

Subgroup analysis	Univariate analysis (unadjusted hazard ratio [95% CI])	Multivariate analysis (adjusted hazard ratio [95% CI])
Overall	1.15 (0.86–1.54)	0.74 (0.55–1.01)
Disease phenotype Crohn's disease Ulcerative colitis	1.17 (0.79–1.72) 1.10 (0.70–1.73)	0.71 (0.48–1.05) 0.81 (0.49–1.33)
Age >60 yr 60 yr or less	0.79 (0.47–1.33) 1.18 (0.82–1.68)	0.54 (0.32–0.92) 0.85 (0.58–1.23)
Index biologic TNFα antagonists Vedolizumab	1.27 (0.93–1.74) 0.68 (0.30–1.55)	0.82 (0.59–1.14) 0.48 (0.19–1.19)
Elixhauser index 0–1 2 or more	0.45 (0.11–1.81) 0.87 (0.64–1.18)	0.42 (0.11–1.64) 0.76 (0.55–1.04)

CI, confidence interval.

On Cox proportional hazard analysis, after adjusting for covariates, including age, sex, race/ethnicity, disease phenotype, index biologic agent, comorbidity burden, abdominal surgery, hospitalization or ED visit, serious infections, and medications, obesity was not associated with an increased risk of serious infections (adjusted HR, 0.74; 95% confidence interval, 0.55–1.01) (Table 3). Similar results were observed on multivariable analysis stratified by disease phenotype, age, type of biologic exposure, and comorbidity burden. Advanced age (60 years or more vs <30 years), Hispanic (vs White), multimorbidity (Elixhauser index, 4 or more vs 0–1 score), recent corticosteroid exposure, opiate use, ED visit, and serious infection in the preceding 12 months were associated with an increased risk of serious infections (Table 3). On *post hoc* analysis, no interaction was observed between obesity and diabetes in modifying the risk of serious infections.

**Table 3.** Multivariable Cox proportional hazard analysis evaluating risk factors for serious infections in biologic-treated patients with inflammatory bowel diseases

Risk factors	Hazard ratio (95% CI)	*P* value
Obese (vs nonobese)	0.74 (0.55–1.01)	0.06
Male (vs female)	0.93 (0.78–1.11)	0.41
Age category <30 yr 30–39 yr 40–59 yr 60 yr or more	1.00 0.89 (0.68–1.18) 0.98 (0.77–1.25) **2.28 (1.75–2.96)**	— 0.43 0.88 **<0.01**
Crohn's disease (vs ulcerative colitis)	0.95 (0.79–1.15)	0.62
Race White African American Hispanic Asian	1.00 1.10 (0.85–1.42) **1.57 (1.17–2.11)** 0.96 (0.57–1.63)	— 0.47 **<0.01** 0.89
Elixhauser index 0–1 2 3 4 or more	1.00 1.21 (0.93–1.56) 1.27 (0.95–1.69) **1.65 (1.28–2.13)**	— 0.15 0.11 **<0.01**
TNFα antagonists (vs vedolizumab)	1.08 (0.83–1.41)	0.58
Corticosteroid use (in preceding 3 mo)	**1.30 (1.07–1.58)**	**<0.01**
Immunomodulator use (in preceding 3 mo)	0.82 (0.62–1.08)	0.17
Opiate use (in preceding 12 mo)	**1.25 (1.03–1.51)**	**0.021**
Hospitalization (in preceding 12 mo)	1.26 (0.99–1.59)	0.06
Emergency department visit (in preceding 12 mo)	**1.48 (1.18–1.85)**	**<0.01**
Abdominal surgery (in preceding 12 mo)	0.77 (0.58–1.03)	0.08
Serious infection (in preceding 12 mo)	**1.80 (1.38–2.33)**	**<0.01**
Diabetes (yes vs no)	**1.40 (1.03–1.92)**	**0.03**
Obesity × diabetes	1.03 (0.53–2.00)	0.93

CI, confidence interval.

Bold entries represent statistically significant risk factors for serious infections.

## DISCUSSION

In this large claims-based study of approximately 6,000 biologic-treated patients with IBD, of whom 8.8% were obese, we observed that obesity is not associated with an increased risk of serious infections, after adjusting for comorbidities. We confirmed prior observations that advanced age, high comorbidity burden, corticosteroid and opiate use, ED visits, and prior infection-related hospitalizations are associated with an increased risk of serious infections in biologic-treated patients. Although obesity has been associated with an increased risk of disease-related complications and impaired treatment response, these findings on safety suggest that obesity may not increase the susceptibility of biologic-treated patients to serious infections. This is a reassuring and very important piece of information because obese patients may require more aggressive immunosuppressive therapy to manage their IBD.

Pathophysiologically, obesity-related immune system dysregulation, decreased cell-mediated immune responses, obesity-related comorbidities, and respiratory dysfunction may predispose obese individuals to infections (7,8,24). Obesity has been consistently associated with an increased risk of nosocomial and surgical site infections in hospitalized patients, but its association with community-acquired infections is inconsistent (24). There have been limited studies on whether obesity may modify the risk of serious infections in patients with IBD. In a propensity score–matched cohort study of hospitalized patients with IBD, obese patients experienced a higher risk of readmission and preventable hospitalizations (3). Although obese patients were significantly more likely to be hospitalized for cardiovascular and respiratory complications as compared to nonobese patients, there was no difference in the risk of infection-related hospitalization between obese vs nonobese patients. Obesity-associated comorbidities, such as diabetes, have also been associated with an increased burden of hospitalization and risk of serious infections (25). Although several registry studies and open-label extension studies have examined risk factors associated with serious infections in biologic-treated patients with IBD, they have not examined obesity as a putative risk factor in univariate or multivariate analysis. Our study provides a critical piece of information, highlighting that obesity may not increase the risk of serious infections in biologic-treated patients with IBD. In fact, we observed a trend toward a lower risk of serious infections in obese patients as compared to nonobese patients. This was in contrast to our *a priori* hypothesis and, as such, difficult to conjecture.

The strengths of our study include: (a) large sample size and high event rates, (b) focus on a contemporary cohort of biologic-treated patients with IBD, and (c) use of validated claims codes for obesity with a high positive predictive value. Our study also has important limitations. First, as an administrative claims-based database study, we did not have access to subjective or objective measures of disease activity or endoscopy reports and did not have accurate details of disease location and behavior, all of which may modify the risk of serious infections. Second, we did not have data on differences in biologic dosing regimens for obese vs nonobese patients. It is likely that obese patients are more likely to require escalation of therapy due to unfavorable pharmacokinetics. However, even if obese patients were more likely to be on higher dose of biologic agents, it is unlikely to have a significantly impacted risk of serious infections. In a recent study, no association was observed between biologic trough concentration and risk of serious infections (26). Third, our definition of obesity relied on body mass index cutoffs. There are other markers of obesity, such as visceral adiposity, which may play a more significant role in IBD outcomes such as risk of infections. Fourth, ideally, infections would be adjudicated by medical record review and microbiology data, but this level of data is unavailable in claims databases. However, our definition of serious infections requiring hospitalization has been validated with a high positive predictive value.

In conclusion, in a contemporary cohort of biologic-treated patients with IBD, obesity was not associated with an increased risk of serious infections. Future studies, relying on more objective measurement of obesity and central adiposity confirming these observations, will provide supportive evidence for aggressive use of immunosuppressive therapy in difficult-to-treat obese patients with IBD.

## CONFLICTS OF INTEREST

**Guarantor of the article:** Siddharth Singh, MD, MS.

**Specific author contributions:** Study concept and design: S.S. and W.J.S. Acquisition of data: H.C.H. Analysis and interpretation of data: S.S. and H.C.H. Drafting the manuscript: S.S. Critical revision of the manuscript for important intellectual content: H.C.H., L.S., N.D.S., and W.J.S. All authors approved the final version of the manuscript.

**Financial support:** This project was supported by the NIH/NIDDK K23DK117058, American College of Gastroenterology Junior Faculty Development Grant, Crohn's and Colitis Foundation Career Development Award, Litwin Pioneers in IBD Award #623346 and IOIBD Operating Grant 2019 (to Siddharth Singh). W.J.S. is partially supported by NIDDK-funded San Diego Digestive Diseases Research Center P30 DK120515. The content is solely the responsibility of the authors and does not necessarily represent the official views of the NIH.

**Potential competing interests:** S.S. reports research grants from AbbVie and Janssen. W.J.S. reports research grants from AbbVie, Abivax, Arena Pharmaceuticals, Boehringer Ingelheim, Celgene, Genentech, Gilead Sciences, GlaxoSmithKline, Janssen, Lilly, Pfizer, Prometheus Biosciences, Seres Therapeutics, Shire, Takeda, and Theravance Biopharma; consulting fees from AbbVie, Abivax, Admirx, Alfasigma, Alimentiv (Robarts Clinical Trials, owned by the Health Academic Research Trust [HART]), Alivio Therapeutics, Allakos, Amgen, Applied Molecular Transport, Arena Pharmaceuticals, Bausch Health (Salix), BeiGene, Bellatrix Pharmaceuticals, Boehringer Ingelheim, Boston Pharmaceuticals, Bristol Meyers Squibb, Celgene, Celltrion, Cellularity, Cosmo Pharmaceuticals, Escalier Biosciences, Equillium, Forbion, Genentech/Roche, Gilead Sciences, Glenmark Pharmaceuticals, Gossamer Bio, Immunic (Vital Therapies), InDex Pharmaceuticals, Intact Therapeutics, Janssen, Kyverna Therapeutics, Landos Biopharma, Lilly, Oppilan Pharma, Otsuka, Pandion Therapeutics, Pfizer, Progenity, Prometheus Biosciences, Protagonist Therapeutics, Provention Bio, Reistone Biopharma, Seres Therapeutics, Shanghai Pharma Biotherapeutics, Shire, Shoreline Biosciences, Sublimity Therapeutics, Surrozen, Takeda, Theravance Biopharma, Thetis Pharmaceuticals, Tillotts Pharma, UCB, Vedanta Biosciences, Ventyx Biosciences, Vimalan Biosciences, Vivelix Pharmaceuticals, Vivreon Biosciences, and Zealand Pharma; and stock or stock options from Allakos, BeiGene, Gossamer Bio, Oppilan Pharma, Prometheus Biosciences, Progenity, Shoreline Biosciences, Ventyx Biosciences, Vimalan Biosciences, and Vivreon Biosciences. Spouse: Iveric Bio—consultant, stock options; Progenity—stock; Oppilan Pharma—consultant, stock options; Prometheus Biosciences—employee, stock, and stock options; Ventyx Biosciences—stock, stock options; and Vimalan Biosciences—stock, stock options. All other authors have no reported conflicts.

**Study Highlights**

**WHAT IS KNOWN**

✓ The prevalence of obesity in patients with inflammatory bowel diseases (IBD) is rising.
✓ Obesity can adversely impact response to biologic therapy in patients with IBD.

**WHAT IS NEW HERE**

✓ Obesity does not increase the risk of serious infections with biologic therapy patients with IBD..
